# RLR-mediated antiviral innate immunity requires oxidative phosphorylation activity

**DOI:** 10.1038/s41598-017-05808-w

**Published:** 2017-07-14

**Authors:** Takuma Yoshizumi, Hiromi Imamura, Tomohiro Taku, Takahiro Kuroki, Atsushi Kawaguchi, Kaori Ishikawa, Kazuto Nakada, Takumi Koshiba

**Affiliations:** 1 Department of Biology, Faculty of Science, Kyushu University, 744 Motooka, Nishi-ku, Fukuoka, 819-0395 Japan; 2 Graduate School of Biostudies, Kyoto University, Yoshida-konoe-cho, Sakyo-ku, Kyoto, 606-8501 Japan; 3 Department of Infection Biology, Faculty of Medicine and Graduate School of Comprehensive Human Sciences, University of Tsukuba, 1-1-1 Tennodai, Tsukuba, Ibaraki 305-8575 Japan; 4 Faculty of Life and Environmental Sciences, University of Tsukuba, 1-1-1 Tennodai, Tsukuba, Ibaraki 305-8572 Japan

## Abstract

Mitochondria act as a platform for antiviral innate immunity, and the immune system depends on activation of the retinoic acid-inducible gene I (RIG-I)-like receptors (RLR) signaling pathway via an adaptor molecule, mitochondrial antiviral signaling. We report that RLR-mediated antiviral innate immunity requires oxidative phosphorylation (OXPHOS) activity, a prominent physiologic function of mitochondria. Cells lacking mitochondrial DNA or mutant cells with respiratory defects exhibited severely impaired virus-induced induction of interferons and proinflammatory cytokines. Recovery of the OXPHOS activity in these mutants, however, re-established RLR-mediated signal transduction. Using *in vivo* approaches, we found that mice with OXPHOS defects were highly susceptible to viral infection and exhibited significant lung inflammation. Studies to elucidate the molecular mechanism of OXPHOS-coupled immune activity revealed that optic atrophy 1, a mediator of mitochondrial fusion, contributes to regulate the antiviral immune response. Our findings provide evidence for functional coordination between RLR-mediated antiviral innate immunity and the mitochondrial energy-generating system in mammals.

## Introduction

Innate immunity is a ubiquitous system that widely protects organisms from infectious pathogens as a front-line host defense mechanism. The immune response is triggered by the recognition of broadly conserved microbial components, known as pathogen-associated molecular patterns, by germline-encoded pattern recognition receptors of the host cells^1^. As an early defense system against RNA viruses in mammals, the innate immune response is precisely controlled by two distinct signal transduction pathways mediated by the pattern recognition receptors Toll-like receptor 3 (TLR-3) and retinoic acid-inducible gene I (RIG-I)-like receptors (RLR) that respond to virus-derived RNAs^2, 3^ (i.e., pathogen-associated molecular patterns). Although the two pathways differ with respect to the initial activation of their downstream effectors, they converge at the point of activation of the transcriptional factors interferon regulatory factor 3 (IRF-3) and nuclear factor κB (NF-κB), which results in the rapid production of type I interferons (IFN-α and -β) and other proinflammatory cytokines to establish adaptive antiviral immunity^4^.

Mitochondria, eukaryotic cell powerhouses, are crucially involved in numerous cellular processes, including apoptosis^5^ and calcium homeostasis^6^. Mitochondria also have a unique role in innate immunity against RNA viruses^7^. Mitochondrial-mediated antiviral immunity depends on activation of the RLR signaling pathway, and mitochondrial antiviral signaling (MAVS), a downstream adaptor of RLR at the mitochondrial outer membrane (MOM), has a key role in the signal transduction^8, 9^. Upon viral infection, MAVS recruits various types of effectors at the MOM, and the orchestrated “MAVS signalosome”, including the mitochondrial membrane potential (Δψ_m_), is the primary unit governing antiviral innate immunity^10, 11^. Although the role of the MAVS signalosome in mitochondria with its dynamic morphologic properties^12^ to provide a molecular platform that facilitates signal transduction is well characterized, insight into how the organelle functions to facilitate antiviral immunity through the activity of oxidative phosphorylation (OXPHOS) has remained unclear.

## Results and Discussion

### Cultured cells rely on mitochondrial respiratory activity

**Figure 1:**
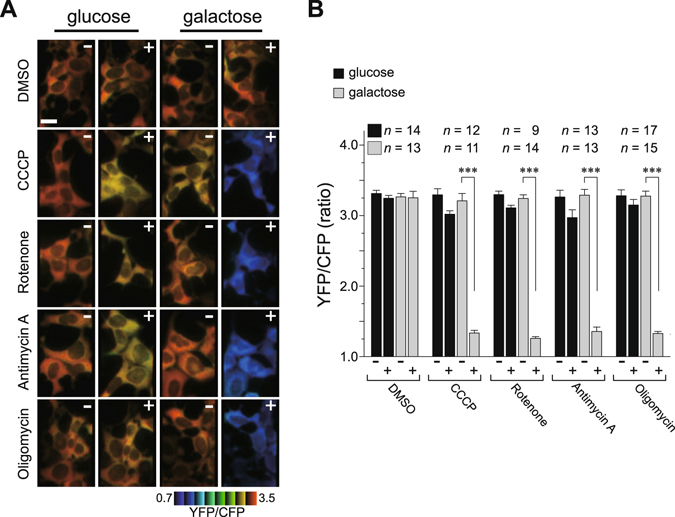
Bioenergetic profiling of cells under oxidative conditions. (**A**) HEK293 cells stably expressing the ATeam1.03 were cultured in customized media containing either galactose (right) or glucose (left) at 37 °C, and then the indicated mitochondrial inhibitors were added to each medium. The time-course of the fluorescence emission ratio (YFP/CFP) was monitored to visualize cytosolic ATP levels in each living cell, and ratiometric pseudocolor images of cells pre- (−) or post-treated (+) with inhibitors are shown. The images were processed in MetaMorph (Molecular Devices), and the blue color indicates less cytoplasmic ATP. Scale bar, 20 μm. (**B**) Quantification of the YFP/CFP ratio calculated from images in (**A**). Number of cells (*n*) used for the quantification is shown at the top of the graph. Error bars indicate SD (Unpaired *t*-test; ***P < 0.001).

To evaluate the functional coordination of mitochondrial-mediated antiviral immunity and OXPHOS activity, we first sought to determine optimal cell culture conditions in which the cellular bioenergetics would rely on mitochondrial respiratory activity. We used a fluorescence resonance energy transfer (FRET)-based assay to visualize metabolized intracellular adenosine 5′-triphosphate (ATP) at the single-cell level in human embryonic kidney 293 (HEK293) cells expressing an ATP probe, ATeam1.03^13^. The biosensor assay performed with cells cultured under our customized medium containing galactose (10 mM) as the carbon source revealed high FRET signal [based on an emission ratio of 527/475 nm (denoted YFP/CFP)] in individual cells [Fig. 1A, galactose panels, (−)], indicating that cells maintained adequate intracellular ATP levels. The intracellular ATP level, however, which affects the YFP/CFP ratio, was dramatically decreased (~2.5-fold) by the addition of electron transport chain (ETC) inhibitors (rotenone and antimycin A), an ATP synthase inhibitor (oligomycin), or a protonophore [carbonyl cyanide *m*-chlorophenylhydrazone (CCCP)] to the media [Fig. 1A, galactose panels, (+), and B], demonstrating that energy production in the cells critically depends on mitochondrial OXPHOS. In contrast, cells maintained in a customized medium containing glucose (10 mM) exhibited no significant decrease in the intracellular ATP level, even after treatment with the pharmacologic drugs (Fig. 1A,B). These results clearly demonstrated that the cellular bioenergetics are less dependent on OXPHOS activity in the presence of glucose, and that cellular ATP production predominantly relies on glycolysis.

### Validation of the RLR signaling pathway under oxidative conditions

**Figure 2:**
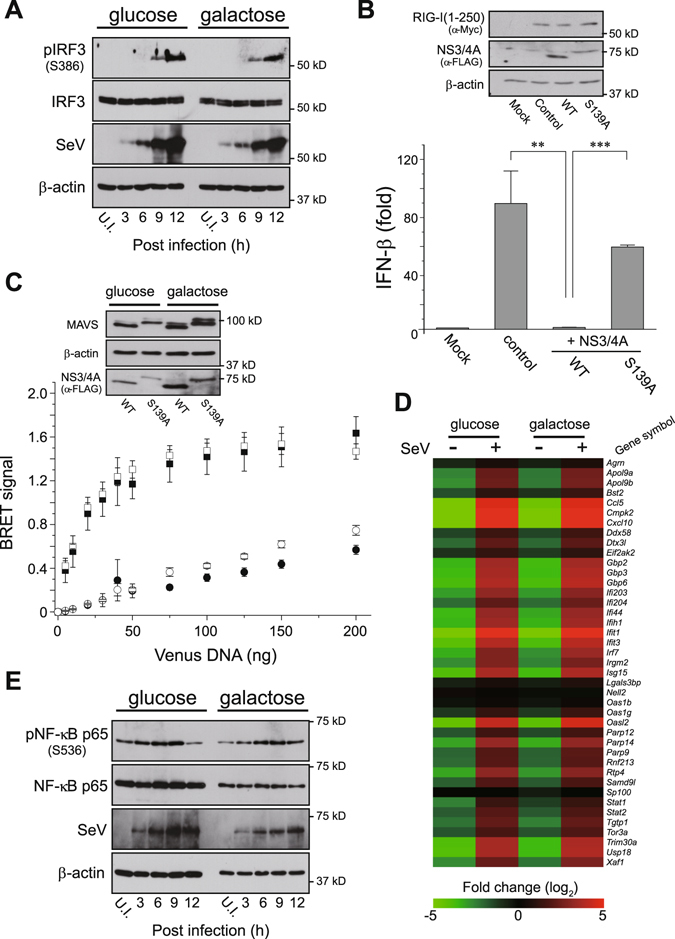
RLR-mediated signal transduction under oxidative conditions. (**A**) The kinetic profile of IRF-3 activation in oxidative or glycolytic medium-cultured HEK293 cells that were challenged with SeV (4 HA units/mL). Each cell lysate was collected at the indicated time-points (3, 6, 9 and 12 h) and analyzed by Western blotting with antibodies against the specific antibody (pIRF-3; phosphorylation of Ser386). Anti-β-actin was used as the loading control and anti-SeV as the infection control. U.I., uninfected. (**B**) Oxidative medium-cultured HEK293 cells were transfected with 50 ng of empty vector (Mock) or expression plasmid for Myc-tagged RIG-I(1–250) (control) together with the IFN-β reporter plasmid. The two right lanes (+NS3/4A) indicate that 100 ng of FLAG-tagged WT or inactive (S139A) NS3/4A serine protease expression plasmids were also co-transfected with the RIG-I(1–250) plasmid. The immunoblot on the top represents an expression profile of Myc-tagged RIG-I(1–250) and FLAG-tagged NS3/4A mutants as well as the loading control of endogenous β-actin. Error bars indicate SD (*n* = 3; Unpaired *t*-test; **P < 0.01 and ***P < 0.001, respectively). (**C**) BRET saturation assay of MAVS oligomerization in glycolytic versus oxidative media. HEK293 cells were co-transfected with 5 ng NLuc-MAVS expression plasmid and increasing amounts (0–200 ng) of Venus-tagged MAVS plasmid along with 200 ng of WT (circles) or S139A (squares) FLAG-tagged NS3/4 A plasmids, and analyzed 24 h later using a BRET saturation assay. Closed and open symbols represent glycolytic and oxidative conditions of HEK293 cells, respectively, and inset blots show Western blots from the BRET saturation point of each curve by immunoblotting with the indicated antibodies. Error bars indicate SEM (*n* = 3). (**D**) Heat maps of microarray analysis. Total RNAs were isolated from glycolytic and oxidative cultured conditions of primary MEFs that were unchallenged (−) or challenged (+) with SeV (30 HA units mL^−1^) for 6 h, and microarray analysis was performed. The heat map was generated by MeV software^42^, and the color indicates the distance from the median of each row. (**E**) Similar to (**A**), except that J774A.1 macrophages were challenged with SeV (2 HA units/mL). U.I., uninfected.

We examined RLR-mediated signal transduction under the galactose-containing oxidative condition. Under oxidative conditions, similar to the glucose condition, ectopic expression of MAVS in HEK293 cells potently activated both IFN-β and NF-κB luciferase reporters in a dose-dependent manner (Supplementary Fig. S1A). The OXPHOS-dependent RLR-mediated signal transduction was also confirmed by stimulating cells transfected with either a plasmid encoding RIG-I(1–250) (Supplementary Fig. S1B) or a synthetic analog of viral double-stranded RNA [dsRNA; poly(I:C)] (Supplementary Fig. S1C and D), both of which are upstream factors of MAVS^3^. The kinetic response of IRF-3 phosphorylation (a hallmark of IRF-3 activation) in cells against infection by Sendai virus (SeV), a negative-stranded RNA virus of the *Paramyxoviridae* family, in the OXPHOS-dependent condition was also similar to that in the glucose condition (Fig. 2A). The RIG-I-mediated activation of the IFN-β reporter in the OXPHOS-dependent condition was sufficiently impaired by co-expression of a hepatitis C virus serine protease NS3/4A (an inhibitor of the RLR signaling pathway)^14, 15^ [Fig. 2B, wild-type (WT)], whereas its inactive mutant (S139A) had no functional effect, indicating that the observed signal transduction occurred via RLR-dependent signaling pathway.

We next analyzed the structural features of MAVS activation accompanied by RLR-mediated signal transduction, homotypic oligomerization at the MOM^11^, when cells were dependent on the oxidative condition. The interaction between ectopically expressed Venus- and NanoLuc luciferase (NLuc)-tagged MAVS with co-expression of the S139A mutant was successfully monitored, and a bioluminescence resonance energy transfer (BRET)-based assay^16^ revealed a hyperbolic saturation curve characteristic of a specific interaction (Fig. 2C, squares). Importantly, the specificity of the observed MAVS-MAVS interaction was verified by the disappearance of the saturated curve when the WT NS3/4A protease was co-expressed in cells due to MAVS cleavage^14^ (Fig. 2C, circles, also see immunoblot). We further verified the antiviral immune response of primary mouse embryonic fibroblasts (MEFs) against SeV infection in the OXPHOS-dependent condition. Gene expression profiling of MEFs showing its dependence on the oxidative condition (Supplementary Fig. S2, upregulation of OXPHOS genes) revealed the expected induction of numerous IFN-stimulated and antiviral-signaling genes in response to the viral infection, similar to that observed in the glucose condition (Fig. 2D). Consistent with the aforementioned assays, we also observed that the antiviral immune response of macrophages cultured under the oxidative conditions was intensively upregulated upon SeV infection (Fig. 2E).

### RLR-mediated antiviral innate immunity requires OXPHOS activity

**Figure 3:**
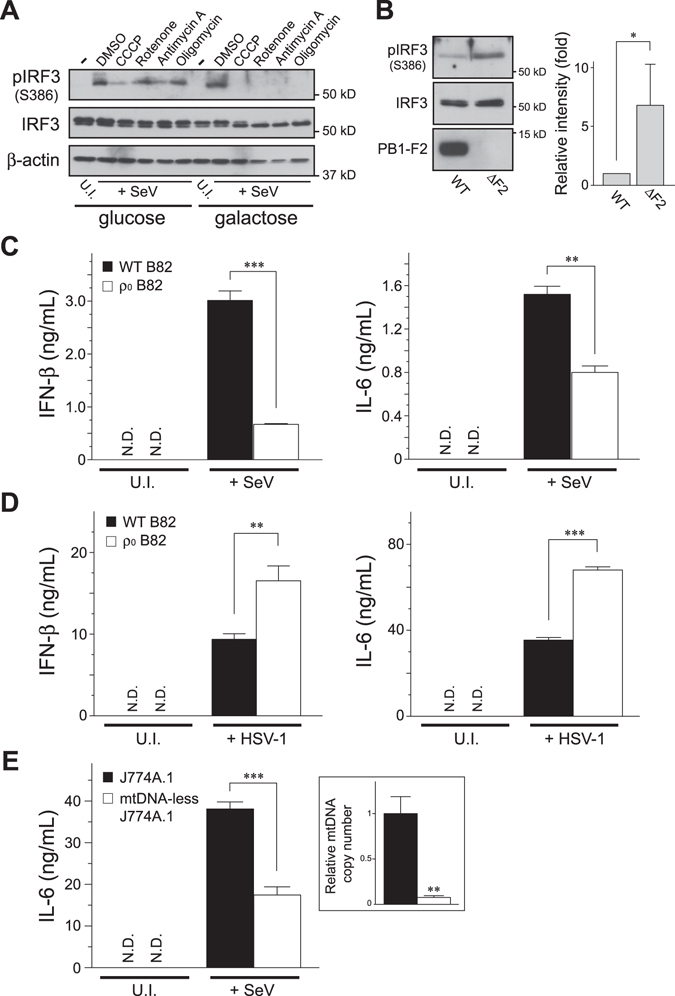
OXPHOS activity couples with the RLR pathway to execute antiviral signal transduction. (**A**) HEK293 cells cultured in glycolytic or oxidative media were infected with SeV (4 HAU/mL) for 2.5 h, and the infected cells were further incubated with the indicated mitochondrial inhibitors for 2.5 h (total 5 h infection). The activation of endogenous IRF-3 was analyzed by Western blotting with antibodies against the specific antibody (pIRF-3; Ser386). Anti-β-actin was used as the loading control. U.I., uninfected. (**B**) Comparison of the IRF-3 activation between cells infected with recombinant influenza A/PR8 viruses (*WT* versus Δ*F2*, each used 2 HAU/mL) cultured under oxidative conditions. The ΔF2 strain is a mutant strain with genetic removal of the *PB1*-*F2* gene from the viral genome^21^. The graph on the right shows the quantification of pIRF-3 bands analyzed by densitometry. Error bars indicate SD (*n* = 3; Unpaired *t*-test; *P < 0.05). (**C**,**D**) The B82 WT cybrids and ρ_0_ cells were infected with (**C**) SeV (4 HAU/mL) for 18 h or (**D**) HSV-1 (1 × 10^5^ PFU) for 24 h, and the cell-free supernatants were analyzed by ELISA to measure the secreted amounts of IFN-β (left panel) and IL-6 (right panel), respectively. Error bars indicate SD (*n* = 3; Unpaired *t*-test; **P < 0.01 and ***P < 0.001, respectively). U.I., uninfected. N.D., not detected. (**E**) Similar to (**C**), except that the mtDNA-less J774A.1 and its parental macrophages were infected with SeV (2 HA units/mL). Inset panel: relative mtDNA copy number was confirmed by qPCR. In (**C**–**E**), cells were maintained in ρ_0_ medium.

Because altering the energy metabolism in cells through the activity of OXPHOS could accomplish the antiviral immune response, we next asked whether arresting OXPHOS activity would disrupt RLR-mediated signal transduction. IRF-3 phosphorylation in cells responding to SeV infection was completely suppressed by the addition of ETC inhibitors to the medium under oxidative conditions (Figs 3A and S3). The functionality of the cells treated with the pharmacologic drugs under these conditions, however, was unaffected when the cells were either stimulated with extracellular poly(I:C) (Supplementary Fig. S4A) or infected with a double-stranded DNA virus, Herpes simplex virus 1 (HSV-1) (Supplementary Fig. S4B), which activates the TLR-3 or cGAS/STING cytosolic DNA sensing pathways, respectively. Influenza A virus (IAV; PR8 strain), an RNA virus of the *Orthomyxoviridae* family, encodes the viral protein PB1-F2 that translocates into mitochondria and induces mitochondrial depolarization^17–20^. Using the viral strain, we observed severe PB1-F2–induced inhibition of RLR signaling in cells that depended on OXPHOS (Fig. 3B, WT); the inhibitory effect was attenuated, however, when we used a recombinant virus with genetic ablation of the PB1-F2 gene (ΔF2)^21^. These observations suggest that the severity of the mitochondrial dysfunction correlates with the severity of the defect in the RLR-induced antiviral response.

We next used a genetic approach to examine whether mitochondrial DNA (mtDNA), which is essential for mitochondrial OXPHOS activity^22^, modulates RLR-mediated signal transduction. Mouse fibrosarcoma B82 cells devoid of mtDNA (ρ_0_ B82)^23^ (Supplementary Fig. S5A) produced less IFN-β and IL-6 against SeV infection, although the immune response was fully rescued in a cell cybrid harboring WT mtDNA (Fig. 3C), despite the fact that both cell types had adequate energy due to the use of a high glucose medium (25 mM) with pyruvate and uridine (Supplementary Fig. S5B) or the same infection level (Supplementary Fig. S5C). Conversely, ρ_0_ B82 cells had substantial antiviral responses against HSV-1 infection (Fig. 3D), consistent with a previous observation^24^. To confirm that these observations were not due to specific characteristics of the cell type we used, we performed the same experiment in HeLa cells lacking mtDNA^25^ (ρ_0_ HeLa), and found that ρ_0_ HeLa cells also exhibited defective antiviral innate immune responses against SeV infection (Supplementary Fig. S5D and E). In addition, transient depletion of mtDNA in macrophages (mtDNA-less J774A.1) treated with rhodamine 6G exhibited an impaired immune response against SeV infection (Fig. 3E). Taken together, these findings indicated that RLR-mediated signal transduction requires OXPHOS activity to execute antiviral immune responses.

### Pathogenic mtDNA mutations cause defects in antiviral innate immunity

**Figure 4:**
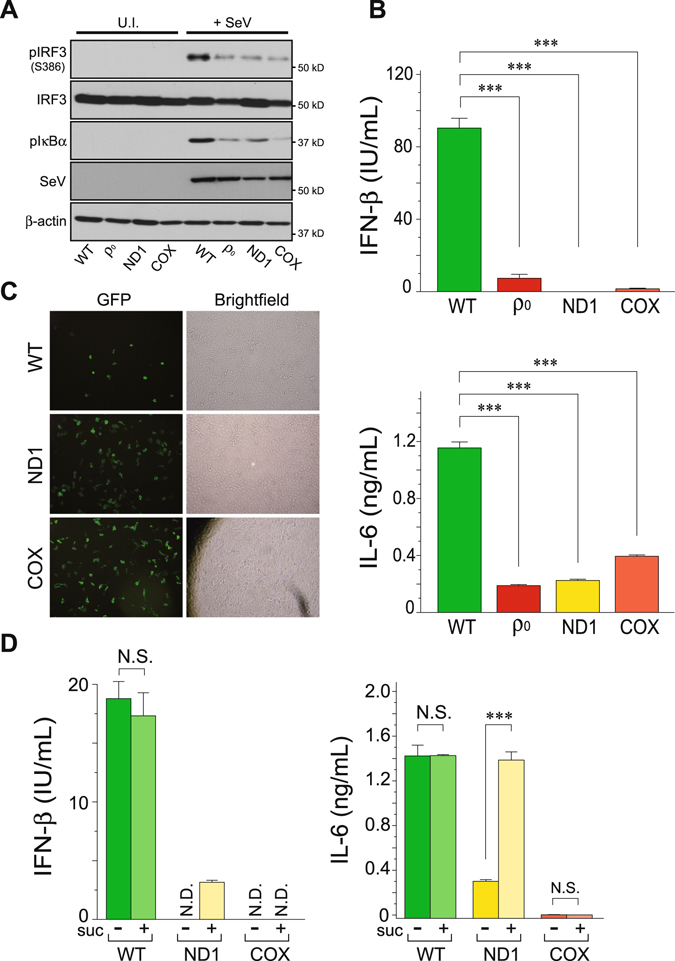
Defects in mtDNA cause malfunction in antiviral innate immunity. (**A**,**B**) The 143B cybrids and ρ_0_ cells were infected with SeV (5 HAU/mL) for 18 h, and (**A**) activation of both IRF-3 and IκBα was analyzed by Western blotting with antibodies against its specific phosphorylated-detection antibodies (Ser386 for IRF-3 and Ser32/36 for IκBα) or (**B**) the cell-free supernatants were analyzed by ELISA to measure the secreted amounts of IFN-β (top panel) and IL-6 (bottom panel). U.I., uninfected. Error bars indicate SD (*n* = 3; Unpaired *t*-test; ***P < 0.001). (**C**) Fluorescence microscopy of 143B cybrid cells infected with IAV-GFP (0.6 HAU/mL) for 24 h. In (**A**–**C**), cells were maintained in ρ_0_ medium. (**D**) Similar to (**B**), except that succinate (suc), the complex II substrate with ADP, was added to the oxidative medium as indicated. Error bars indicate SD (*n* = 3; Unpaired *t*-test; ***P < 0.001; N.D., not detected; N.S., not significant).

The importance of mtDNA in the regulation of RLR-mediated signal transduction prompted us to explore the phenotypic effects of pathogenic mtDNA mutations on antiviral immunity. We used human osteosarcoma cybrid 143B cells harboring homoplasmic pathogenic mtDNA mutations: the ND1 cybrid^26^ (complex I dysfunction), which is associated with Leber hereditary optic neuropathy, carrying a missense mutation at 3460/ND1 (Supplementary Fig. S6A); and the COX cybrid^27^ (missing complex IV), which is associated with mitochondrial encephalopathy, carrying a stop codon mutation at 6930/COXI (Supplementary Fig. S6B), both of which exhibit severe respiratory defects. As described, ρ_0_ cells from 143B cell lines also exhibited an impaired immune response against SeV infection (Fig. 4A,B). Remarkably, activation of the RLR-mediated antiviral signaling in the mutant cells (ND1 and COX) was severely disrupted (Fig. 4A,B) despite the indistinguishable MAVS pattern and energy supply (Supplementary Fig. S7A, B and C). Consistent with these results, both pathogenic mutants were more susceptible to viral infection (recombinant IAV-expressing GFP)^28^ than WT cells (Fig. 4C), due to defective mitochondrial-mediated immunity. Most importantly, the immunodeficiency observed in the ND1 cybrid, but not in the COX cybrid, was significantly recovered by the addition of succinate, a complex II substrate, to the media, suggesting that OXPHOS activity in ND1 mutant cells was driven by the substrate through complex II, which consequently restored its phenotype (Fig. 4D). Together, these results indicated that cells with defective OXPHOS activity due to mtDNA mutations have severely impaired RLR-mediated antiviral signaling.

### Trans-mitochondrial mice (Mito-miceΔ) are susceptible to IAV infection

**Figure 5:**
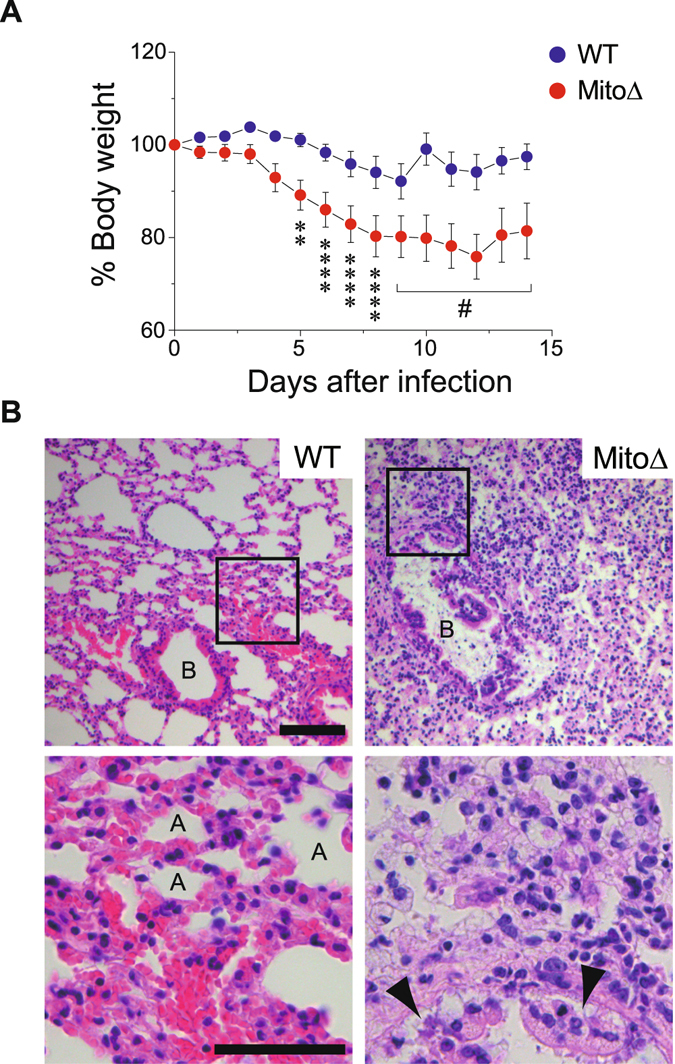
OXPHOS-defective mice are highly susceptible to viral-infection. (**A**) Mito-miceΔ (*n* = 7) or WT mice (*n* = 9) were challenged with IAV (1 × 10^3^ PFU) and mouse weight loss was monitored for 14 days. In the graph, the percentage change from the initial weight of the mice is shown. Error bars indicate SEM [Dunnett’s test; **P < 0.01 and ****P < 0.0001 (versus day 0), respectively)]. ^#^Dunnett’s test could not be applied because dead mice appeared from day 9. (**B**) In a separate experiment, the lungs were obtained from each infected mouse on day 8 post-infection, sectioned, and analyzed for histopathology following staining with hematoxylin and eosin. Enlarged boxes are depicted in lower images (400× magnification). Labels **A** and **B** in the images indicate alveoli and bronchus, respectively, and arrowheads indicate desquamation of the bronchial epithelium. Scale bars, 100 μm (top) and 50 μm (bottom), respectively.

To evaluate whether our *in vitro* observations were physiologically relevant, we investigated the relationship between OXPHOS and antiviral innate immunity *in vivo*. We used 28-week-old trans-mitochondrial mice carrying mtDNA with a large-scale deletion (Mito-miceΔ)^29^ and challenged the mice with the IAV PR8 strain. Mito-miceΔ in a resting state had high blood lactate levels (7.2 ± 2 mmol/L) relative to those in WT mice (3.2 ± 0.4 mmol/L), showing lactic acidosis in the mutant mice. Although the mortality of Mito-miceΔ (*n* = 7) infected with IAV was not much worse than that of WT mice (*n* = 9) (Supplementary Fig. S8A), the Mito-miceΔ exhibited significant weight loss during the monitoring period (Fig. 5A; 17% of Mito-miceΔ versus 4% of WT on day 7). Given the higher pathogenicity in Mito-miceΔ following IAV-infection, we examined the physiologic effects of the infection on the lung tissue. In the lungs of WT mice infected with IAV, mild hyperemia and perivascular inflammatory cells infiltration around the bronchus were minimally observed, but few alveoli were injured by the infection (Fig. 5B, left panels). On the other hand, Mito-miceΔ infected with the virus had significant inflammation in whole lung tissues with desquamation of the bronchial epithelium (Fig. 5B, right panels, see arrowheads) and alveolar pneumocyte hyperplasia. Notably, virus-induced expression of endogenous *IFN-*β was impaired in the lungs of the Mito-miceΔ (Supplementary Fig. S8B), explaining the histopathologic observation of enhanced pathogenicity. We thus concluded that defective OXPHOS enhances the susceptibility to IAV infection *in vivo*, emphasizing the importance of mitochondrial respiration for modulating innate immunity.

### OPA1-mediated execution of antiviral innate immunity

**Figure 6:**
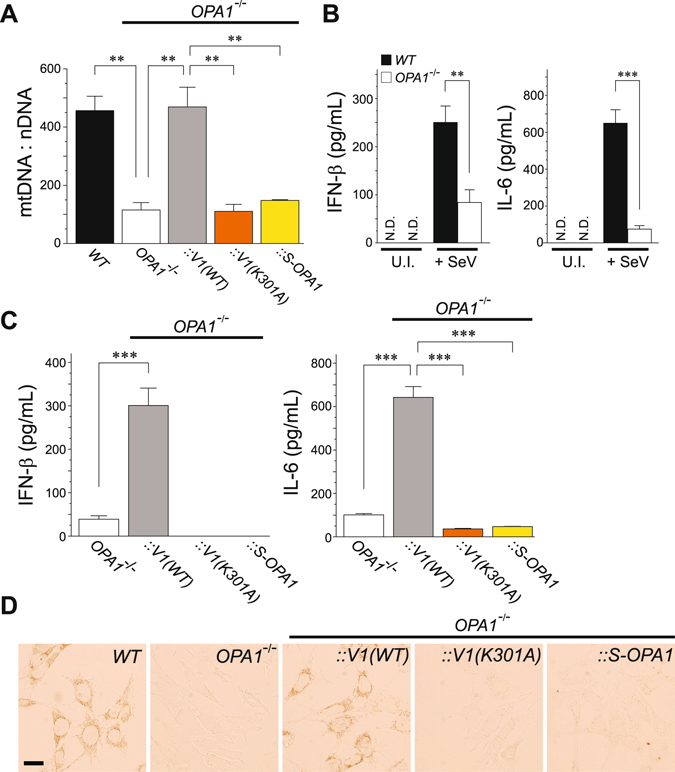
OPA1 contributes to mitochondrial-mediated antiviral signaling through stabilizing mtDNA. (**A**) Analysis of mtDNA copy number per nuclear DNA in WT and mutant MEFs. Restoration of mtDNA in *OPA1* null cells was measured in mutant cells infected with a retrovirus-expressing variant 1 WT *OPA1* [*V1(WT)*], its K301A mutant [*V1(K301A)*], and short isoform *OPA1* (*S-OPA1*). Error bars indicate SEM (*n* = 3; Unpaired *t*-test; **P < 0.01). (**B**) SeV-induced antiviral innate immune response in *OPA1*-null MEFs. The *WT* and *OPA1*-null MEFs were either uninfected (U.I.) or infected with SeV (4 HAU/mL) for 18 h, and the cell-free supernatants were analyzed by ELISA to measure the secreted amounts of IFN-β (left panel) and IL-6 (right panel). Error bars indicate SD (*n* = 3; Unpaired *t*-test; **P < 0.01 and ***P < 0.001, respectively). N.D., not detected. (**C**) Similar to (**B**), except that the immune response in mutant *OPA1* MEFs was monitored. Error bars indicate SD (*n* = 3; Unpaired *t*-test; ***P < 0.001). (**D**) Cytochemical analysis of COX activity. Cells expressing COX activity were indicated by a brown color. Scale bar, 20 μm.

To elucidate the molecular basis of OXPHOS activity coupled with the RLR signaling pathway, we speculated that optic atrophy 1 (OPA1), a mediator of mitochondrial fusion^30^, has a role in antiviral innate immunity^12^ because of its stabilizing effects on mtDNA^31^ and the cristae structure^32, 33^, both of which contribute to regulating mitochondrial respiration. As reported previously, we observed that *OPA1* ^−/−^ cells contained less mtDNA (Fig. 6A), completely lacked mitochondrial fusion (Supplementary Fig. S9A), and had a disrupted cristae morphology (Supplementary Fig. S9B). These phenotypic defects were restored when a *WT OPA1* isoform gene (L-OPA1; variant 1), but not an inactive mutant *K301A* or short isoform (*S-OPA1*), was re-introduced into null cells. Strikingly, although the *OPA1* ^−/−^ cells exhibited severely impaired SeV-induced production of IFN-β and IL-6 (Fig. 6B), the immune responses were substantially restored by recovery of the mtDNA level in rescued cells [Figs 6A,C and S9C; *V1(WT)*]. Given that OPA1 is important for mtDNA stability and the antiviral immune response, we used histochemical staining for cytochrome *c* oxidase (complex IV) activity to directly assess the OPA1 function involved in mitochondrial respiration. As expected, the COX staining pattern of the *V1(WT)*-rescued *OPA1* ^−/−^ cells showed full recovery of the respiration defect, whereas *K301A* and *S-OPA1* failed to rescue the COX activities (Fig. 6D). Taken together, these results indicate that functional actions of OPA1 are linked to the improvement of OXPHOS activity and the induction of mitochondrial-mediated antiviral innate immunity.

The findings of the present study using both *in vitro* and *in vivo* approaches provided new insight into how mitochondria facilitate antiviral immunity through OXPHOS activity. Mitochondria are believed to have evolved from organisms such as α-proteobacterium, and the discovery of their role in host-cell defense was unexpected. It is not surprising, however, that cells gaining respiratory function through a symbion could greatly advance not only through enhanced energy production, but also through safeguarded host defense against other infectious pathogens, especially in vertebrates.

## Materials and Methods

### Reagents

CCCP, rotenone, oligomycin, and antimycin A were purchased from Sigma-Aldrich (St. Louis, MO). Cytochrome *c* from horse heart was supplied by Nacalai Tesque Inc. (Kyoto, Japan) and 3,3′-diaminobenzidine was purchased from Tokyo Chemical Industry Co. Ltd. (Tokyo, Japan). Furimazine was supplied by Promega (Madison, WI) and poly(I:C) was purchased from InvivoGen (San Diego, CA). Enzyme-linked immunosorbent assay (ELISA) kits for human and mouse IFN-β were supplied by Kamakura Techno-Science Inc. (Kanagawa, Japan) and PBL Assay Science (Piscataway, NJ), respectively. All other reagents were of biochemical research grade.

### Antibodies

A polyclonal antibody against IRF-3 (FL-425; 1:2000) and monoclonal antibody against β-actin (C4; 1:4000) were purchased from Santa Cruz Biotechnology (Dallas, TX), and the rabbit monoclonal antibody against phosphorylated IRF-3 (pIRF-3) Ser^386^ (EPR2346; 1:2000) and polyclonal antibody against MAVS (ab25084; 1:1000) were from Abcam (Cambridge, MA). Monoclonal antibodies against Myc (9E10; 1;1000) and FLAG (M2; 1:1000) were obtained from Covance (Princeton, NJ) and Sigma-Aldrich, respectively. Monoclonal antibodies against phosphorylated IκBα (pIκBα) Ser^32/36^ (5A5; 1:1000), phosphorylated NF-κB p65 (pNF-κB p65) Ser^536^ (93H1; 1:2000) and NF-κB p65 (D14E12; 1:3000) were supplied by Cell Signaling Technology (Danvers, MA), and the monoclonal antibody against OPA1 (1:2000) was from BD Biosciences (San Jose, CA). The anti-human and anti-mouse IL-6 antibodies used for ELISA were obtained from eBioscience, and a polyclonal antibody against SeV (PD029; 1:1000) was purchased from MBL (Nagoya, Japan). Monoclonal antibodies against mtCO1 (1D6E1A8; 1:1000) and mitochondrial heat shock protein 70 (mtHsp70; 1:2000) were purchased from Molecular Probes/Invitrogen (Carlsbad, CA) and Affinity BioReagents (Golden, CO), respectively. The Alexa Fluor 488–conjugated polyclonal antibody against rabbit immunoglobulin G (IgG) and Alexa Fluor 568–conjugated polyclonal antibody against rabbit IgG (1:500) were obtained from Molecular Probes-Invitrogen, and the Cy3-conjugated sheep anti-mouse IgG (1:1000) monoclonal antibody was purchased from Jackson ImmunoResearch Laboratories (West Grove, PA). A monoclonal antibody against PB1-F2 (A/PR8 strain; 1:10) was a kind gift from Viktor Wixler (Münster University Hospital Medical School).

### Plasmids

Plasmids encoding human MAVS variants, hRIG-I(1–250), NS3/4A variants, and the ATeam1.03 were described previously^13, 16, 20, 34^. Plasmid encoding rat OPA1 was a gift from Naotada Ishihara (Kurume University, Japan). To generate the retroviral expression constructs, each cDNA was recloned into the retroviral vector pMXs-Puro (Cell Biolabs, San Diego, CA). The retroviral expression vectors were then transfected into the platinum packaging cell lines (Cell Biolabs), and the retroviral supernatant was harvested 48 h post-transfection and used to infect cells.

### Cell lines and viruses

HEK293, MEF, and J774A.1 cells were maintained in Dulbecco’s modified Eagle medium (high glucose; 4,500 mg/L; GIBCO BRL) supplemented with 1% GlutaMAX, penicillin (100 U/mL)-streptomycin (100 μg/mL), and 10% fetal bovine serum at 5% CO_2_ and 37 °C. The ρ_0_ cells (lacking mtDNA) from HeLa, B82 (mouse fibrosarcoma), and 143B (human osteosarcoma) and its WT or mutant mtDNA cybrids were similarly maintained, except with the addition of sodium pyruvate (110 mg/L) and uridine (50 μg/mL; ρ_0_ medium). The mtDNA-less J774A.1 macrophages were pretreated with rhodamine 6 G (0.06 μg/mL in ρ_0_ medium) for 48 h to transiently eliminate endogenous mtDNA. For glucose-free culture experiments, glycolytic (glucose) and oxidative (galactose) media were customized according to previously published protocols^35^ with slight modifications; the glycolytic medium was distinct from the high glucose medium used to maintain the cultures. The customized medium was based on glucose and glutamine-free Dulbecco’s modified Eagle medium (GIBCO; A14430–01) externally supplemented with 10% dialyzed fetal bovine serum (GIBCO; 26400–044), 2% GlutaMAX, penicillin (100 U/mL)-streptomycin (100 μg/mL), and either 10 mM glucose (glycolytic) or 10 mM galactose (oxidative), respectively. Sendai virus Cantell strain was purchased from the American Type Culture Collection, and the recombinant influenza viruses A/PR8 (ΔF2)^21^ and IAV-GFP^28^ were a generous gift from Jonathan A. McCullers (St. Jude Children’s Research Hospital) and Adolfo García-Sastre (Icahn School of Medicine at Mount Sinai), respectively.

### Viral infection *in vivo*

Twenty eight-week-old C57BL/6 mice were anesthetized with isoflurane and inoculated intranasally with 50 μL containing 1 × 10^3^ PFU of A/PR8. One group of mice was mock-infected with sterile phosphate buffered saline (PBS) as a control. Mice were observed daily for weight loss and mortality for 14 days. For histopathologic analysis, dissected lung tissues were fixed in 3.5% formaldehyde and paraffin-embedded. Tissues were sliced into 6-μm-thick paraffin sections and stained with hematoxylin and eosin. To determine the level of *IFN-*β mRNA, mice were euthanized 4 days post-infection, and the total RNAs were purified from 30 mg of lung tissue homogenate using an RNeasy Kit (Qiagen). All animal experiments were carried out in a humane manner after receiving approval from the Institutional Animal Care and Use Committee of the University of Tsukuba, and in accordance with the regulation for Animal Experiments in University of Tsukuba and Fundamental Guideline for Proper Conduct of Animal Experiment and Related Activities in Academic Research Institutions under the jurisdiction of the Ministry of Education, Culture, Sports, Science, and Technology.

### Luciferase assays

HEK293 cells were plated in 24-well plates (2.5 × 10^5^ cells per well) in the customized media. The following day, the cells were co-transfected with 100 ng luciferase reporter plasmid (p125luc or pELAM), 2.5 ng *Renilla* luciferase internal control vector phRL-TK (Promega), and each of the indicated expression plasmids with Lipofectamine 2000 reagent (Invitrogen). Empty vector [pcDNA3.1(−)] was used to maintain equivalent amounts of DNA in each well. Cells were harvested 24 h after transfection and analyzed by a dual-luciferase reporter assay on the GloMax 20/20n luminometer (Promega). Each experiment was repeated at least three times.

### BRET assay

All BRET saturation assays were performed as previously described^16^ with slight modifications. In brief, HEK293 cells (5 × 10^5^ cells per well) cultured under customized media were co-transfected with a constant amount (5 ng) of NLuc-tagged MAVS plasmid and increasing amounts of Venus-tagged MAVS constructs using Lipofectamine 2000 reagent. Empty vector [pcDNA3.1(−)] was used to maintain equivalent amounts of DNA in each well. The cells were harvested at 21 h post-transfection and transferred to each well of white 96-well microplates. NLuc substrate (furimazine; 5 μM) was added, and the plates were analyzed via a BRET saturation assay using a Flexstation 3 Microplate Reader (Molecular Devices, Sunnyvale, CA) at 37 °C.

### ELISA

Measurements of species-specific IFN-β and IL-6 production were performed as described previously^20, 36^.

### Microarray analysis

Total RNA was isolated from each cultured condition of primary MEFs using TRIzol Reagent (Invitrogen) and purified using SV Total RNA Isolation System (Promega) according to the manufacturer’s protocol. RNA samples were quantified using an ND-1000 spectrophotometer (NanoDrop Technologies, Wilmington, DE) and the quality was confirmed with an Experion System (Bio-Rad Laboratories, Hercules, CA). The cRNA was amplified, labeled (Low Input Quick Amp Labeling Kit), and hybridized to the SurePrint G3 Mouse Gene Expression 8 × 60 K microarray according to the manufacturer’s instructions (Agilent Technologies, Santa Clara, CA). All hybridized microarray slides were scanned by an Agilent scanner (Agilent Technologies). Relative hybridization intensities and background hybridization values were calculated using Agilent Feature Extraction Software (9.5.1.1). Raw signal intensities and flags for each probe were calculated from the hybridization intensities (gProcessedSignal), and spot information (gIsSaturated, etc.), according to the procedures recommended by Agilent. The raw signal intensities of four samples were normalized by a quantile algorithm with the ‘preprocessCore’ library package^37^ of the Bioconductor software^38^. To identify upregulated or downregulated genes, we calculated the Z-scores^39^ and ratios (non-log scaled fold-change) from the normalized signal intensities of each probe to compare between control and experiment samples, and established criteria for regulated genes: Z-score ≥2.0 and ratio ≥1.5-fold for upregulated genes, and Z-score ≤−2.0 and ratio ≤0.66 for downregulated genes, respectively.

### Visualization of cytoplasmic ATP in living cells

Imaging of cytoplasmic ATP levels in living cells was performed as previously described^13, 40^. Briefly, cells expressing ATeam1.03 were plated on collagen-coated 35-mm glass-bottom dishes (MatTek, Ashland, MA). The following day, the cells were washed once with the customized media and the cells were subjected to ATP imaging. Observations of cells that were maintained at 5% CO_2_ and 37 °C using a stage-top incubator (Tokai Hit, Shizuoka, Japan) were performed using a Nikon ECLIPSE Ti-E microscope with an oil-immersion type CFI Plan Apo VC 60x lens (Nikon Instruments Inc, Tokyo, Japan). The filters, 438/24-nm BrightLine single-band bandpass filter (FF01–438/24–25), 458-nm edge BrightLine single-edge dichroic mirror (FF458-Di02), and two emission filters (FF01–483/32 and FF01–542/27) used for dual-emission ratio imaging of ATeam1.03 were obtained from Semrock Inc. (Rochester, NY). Fluorescence emission from the ATeam1.03 was imaged by altering the emission filters with a filter changer and a scientific CMOS camera (Zyla 4.2, Andor Technology), and the imaging analysis was performed using MetaMorph (Molecular Devices). The YFP/CFP emission ratio was calculated by dividing the YFP intensity by the CFP intensity of each cell.

### Confocal microscopy

Cells were plated on coverslips in 12-well plates (1.5 × 10^5^ cells per well). The following day, cells were fixed with 4% paraformaldehyde for 10 min, permeabilized with 0.2% Triton X-100 in PBS (pH 7.4), and blocked with 5% bovine calf serum. Endogenous MAVS was detected with the polyclonal primary and AlexaFluor488 secondary antibodies, and mitochondria were stained with anti-mtHsp70 primary antibody followed by the Cy3-conjugated secondary antibody. Cells were imaged with the C2^+^ confocal microscope (Nikon Instruments Inc).

### Electron microscopy analysis

For electron microscopy, cells were fixed in 100 mM cacodylate buffer (pH 7.4) containing 2% paraformaldehyde and 2% glutaraldehyde (GA) for 1 h, washed and fixed again with 100 mM cacodylate buffer containing 2% GA overnight at 4 °C. After fixation, the cells were washed four times with 100 mM cacodylate buffer for 20 min each, and postfixed with 2% osmium tetroxide (OsO_4_) in cacodylate buffer at 4 °C for 1 h. Cells were then dehydrated in a graded series of ethanol (50%, 70%, 90%, and 100%), embedded in resin (Quetol 812, Nisshin EM Co., Japan), and polymerized at 60 °C for 48 h. The polymerized resins were cut in ultra-thin sections at 70 nm with an LEICA UTC ultramicrotome, mounted on copper grids, and stained with 2% uranyl acetate and lead solution (Sigma-Aldrich). Images were collected with a transmission electron microscope (JEM-1400Plus; JEOL Ltd, Japan) operating at 80 kV and equipped with a CCD camera (VELETA; Olympus Soft Imaging Solutions GmbH, Germany).

### Quantification of mtDNA

Total genomic DNA was extracted and purified from cells, and diluted to 5 ng/μL. To quantify the amount of mtDNA per nuclear DNA, quantitative real-time polymerase chain reaction (qPCR) was performed using FastStart Essential DNA Green Master (Roche). Quantification of relative copy number differences was performed based on the difference in the threshold amplification between mtDNA and nuclear DNA [ΔΔC(t) method]. We used the following sets of primers for mtDNA: TK702 (5′-cctatcacccttgccatcat, forward) and TK703 (5′-gaggctgttgcttgtgtgac, reverse); and for nuclear DNA: TK704 (5′-atggaaagcctgccatcatg, forward) and TK705 (5′-tccttgttgttcagcatcac, reverse).

### Cytochemical analysis of cytochrome *c* oxidase (COX) activity

COX staining was performed as previously described^41^ with slight modifications. MEFs plated on 18-mm coverslips were fixed with 2% paraformaldehyde in PBS for 15 min and washed twice with PBS for 5 min each. To visualize COX activity, cells were stained with 100 mM phosphate buffer (pH 7.4) containing 0.6 mg/mL 3′,3′-diaminobenzidine, 0.3 mg/mL cytochrome *c*, and 45 mg/mL sucrose at 37 °C for 2 h. Cells were then washed twice with 0.1 M Tris-HCl buffer (pH 8.0) for 5 min each and analyzed by microscopy.

### Statistical analysis

An analysis of variance test (GraphPad QuickCalcs) was used for the statistical analyses. A *P*-value of less than 0.05 was considered statistically significant.

## Electronic supplementary material

Supplementary Information
