# Supplementary material for: RLR-mediated antiviral innate immunity requires oxidative phosphorylation activity
Source: Sci Rep. 2017 Jul 14;7:5379. doi: 10.1038/s41598-017-05808-w (PMC5511143; doi:10.1038/s41598-017-05808-w)
Supplement: Supplementary file 1 — Supplementary Information [file 41598_2017_5808_MOESM1_ESM.pdf]

Supplementary Information

RLR-mediated antiviral innate immunity requires oxidative phosphorylation activity

Takuma Yoshizumi, Hiromi Imamura, Tomohiro Taku, Takahiro Kuroki, Atsushi Kawaguchi, Kaori Ishikawa, Kazuto Nakada, and Takumi Koshiba

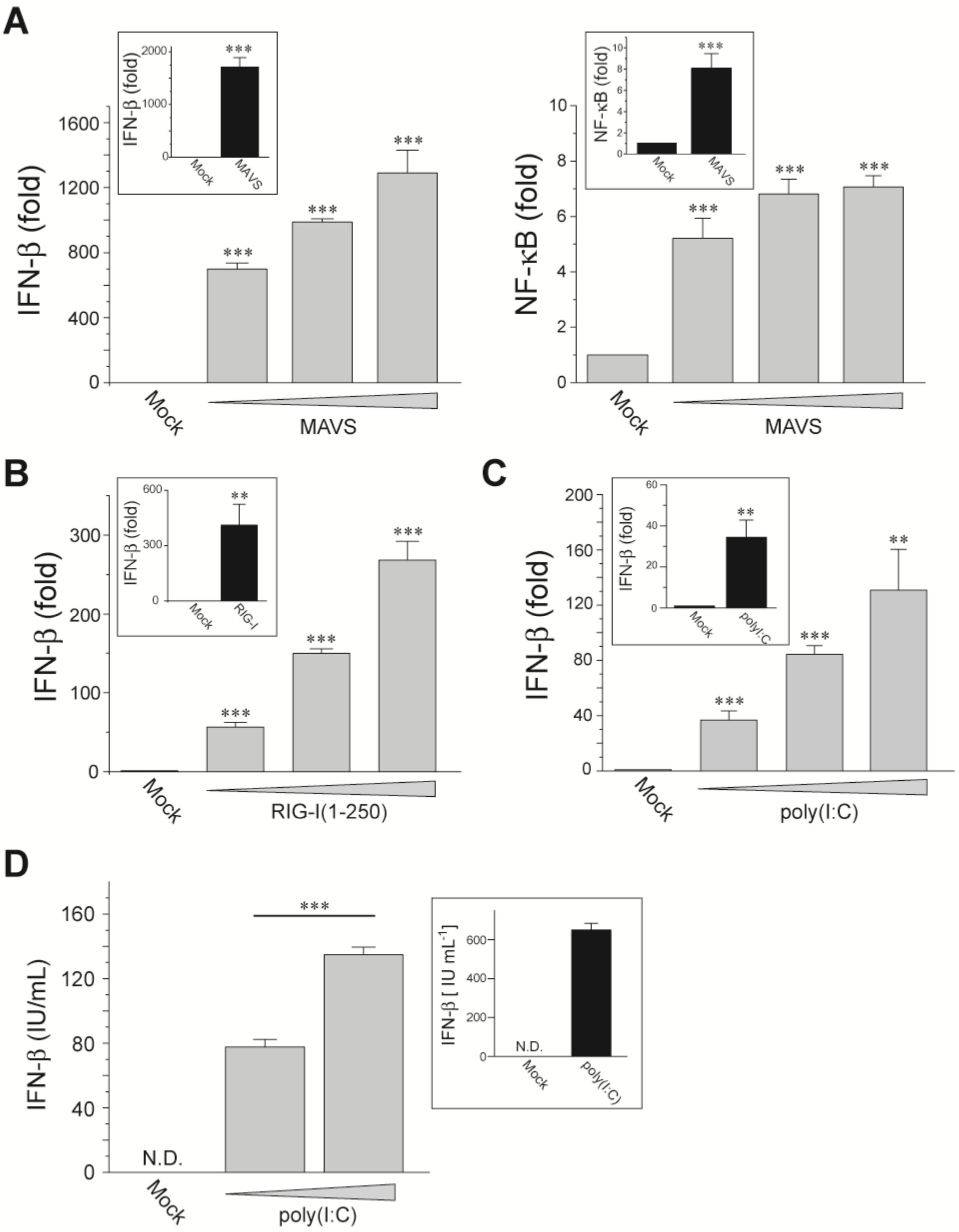

Supplementary Figure S1. RLR-mediated signal transduction in HEK293 cells under oxidative conditions. (**A**) Galactose-cultured HEK293 cells were transfected with empty

vector (Mock) or increasing amounts (10, 20, 50 ng) of plasmids encoding MAVS, and with either IFN-β (left) or NF-κB (right) reporter plasmids. The transfected cells were analyzed 24 h later for reporter gene-dependent luciferase activities. Inset panel: glucose-cultured HEK293 cells (black bars) were also tested as the control (50 ng MAVS plasmids). Error bars indicate SD (n = 3; Unpaired t-test; ***$P < 0.001$). (**B**, **C**) Similar to (A), except that HEK293 cells were transfected with either (B) RIG-I(1-250) or (C) poly(I:C) in a dose-dependent manner (10, 20, 50 ng) together with the IFN-β reporter plasmid. Inset panel: glucose-cultured HEK293 cells (black bars) were also tested as the control. Error bars indicate SD (n = 3; Unpaired t-test; **$P < 0.01$ and ***$P < 0.001$, respectively). (**D**) Similar to (C), except that HEK293 cells were transfected with either empty (Mock) or poly(I:C) (5 or 10 μg), and the cell-free supernatant was collected for measurement of IFN-β production by ELISA. Inset panel: glucose-cultured HEK293 cells (black bar) were also tested as the control [10 μg of poly(I:C)]. Error bars indicate SD (n = 3; Unpaired t-test; ***$P < 0.001$). N.D., not detected in the experiment.

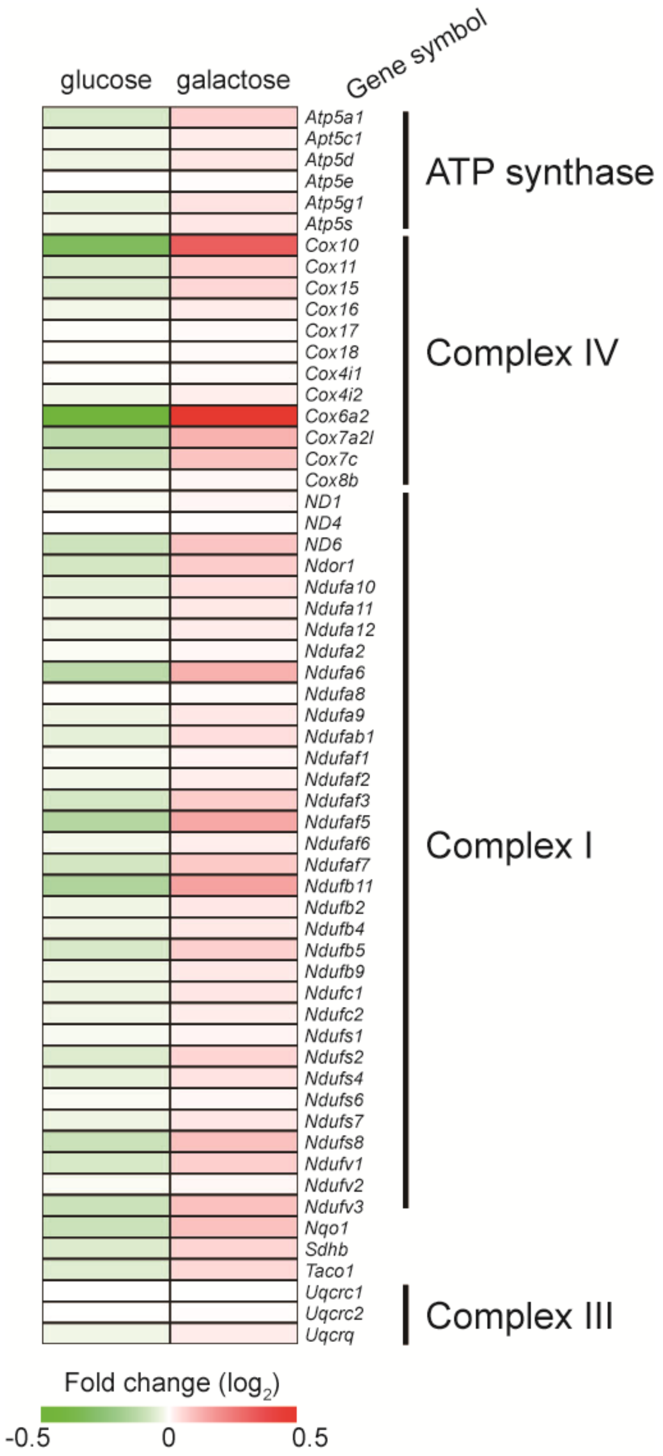

Supplementary Figure S2. Heat map of primary MEFs cultured under glycolytic or oxidative conditions. Total RNAs were isolated from each cultured condition of primary MEFs and microarray analysis was performed. The heat map compared the expression profile of OXPHOS-related genes, and was generated by MeV software[42]. The color indicates the distance from the median of each row.

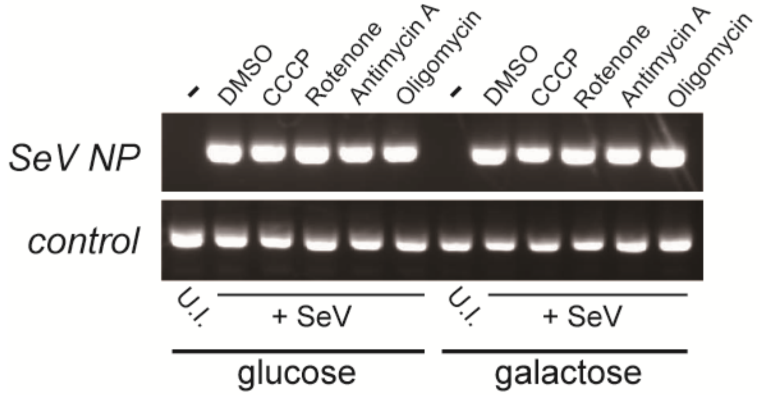

Supplementary Figure S3. Comparison of the SeV-infection level between cells treated with mitochondrial inhibitors. HEK293 cells cultured in glycolytic or oxidative media (incubated with indicated mitochondrial inhibitors) were infected with SeV (4 HAU/mL) for 5 h, and total RNAs from cells were analyzed by RT-PCR to detect viral infection. A region of the SeV *NP gene* (138-386) was amplified and *CLS1* was used as a loading control. U.I., uninfected.

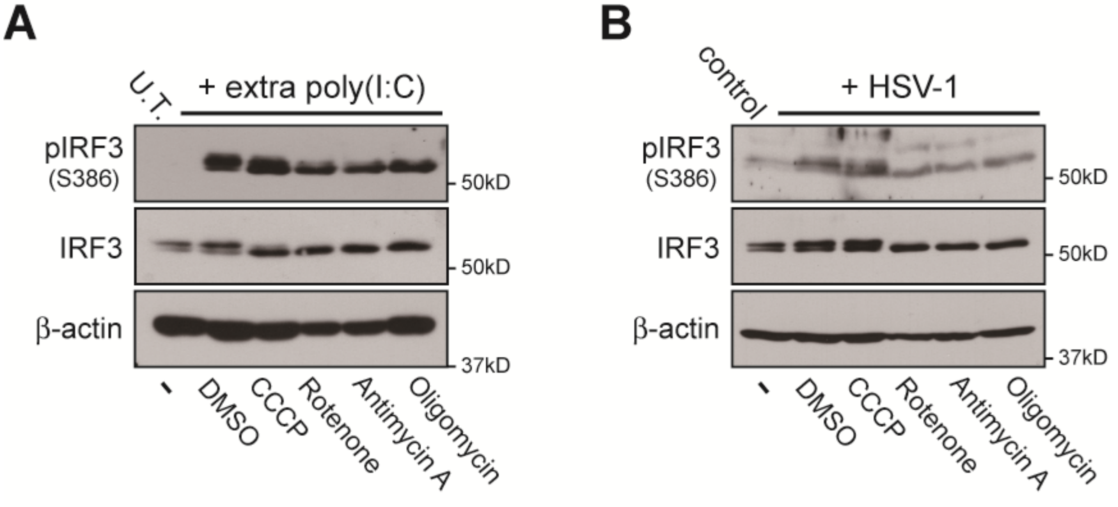

Supplementary Figure S4. TLR-3 or cGAS/STING pathways were not affected in cells treated with mitochondrial inhibitors. (**A**) HEK293 cells stably expressing TLR-3 and cultured in oxidative medium were stimulated with extracellular poly(I:C) (5 μg) and the indicated mitochondrial inhibitors for 5 h. Activation of endogenous IRF-3 was analyzed by Western blotting with antibodies against the specific antibody (pIRF-3; phosphorylation of Ser386). Anti-β-actin was used as the loading control. U.T., untreated. (**B**) Similar to (A), except that HEK293 cells stably expressing both cGAS and STING were infected with HSV-1 (5×10^4 PFU, total 12 h infection).

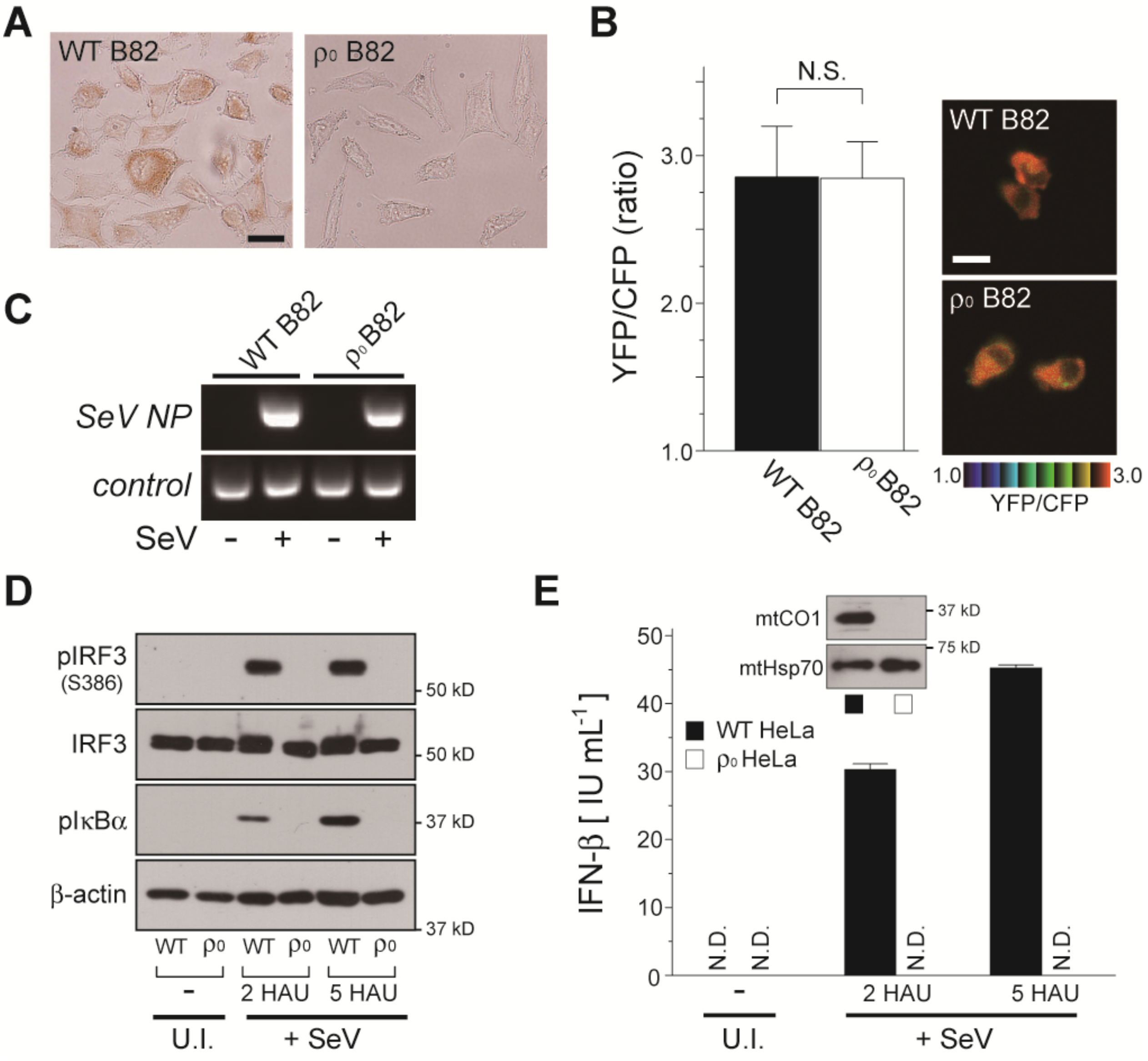

Supplementary Figure S5. Defective RLR pathway in mtDNA-depleted cells. (**A**) Comparison of COX activity between B82 WT cybrids and ρ_0 cells. Cells expressing COX activity were indicated by a brown color. Scale bar, 20 µm. (**B**) The B82 WT cybrids and ρ_0 cells were transfected with ATeam1.03-expressing plasmid. Cytoplasmic ATP levels in the cells were monitored 24 h post-transfection. The graph on the left shows quantification of the YFP/CFP ratio calculated from analyzed images (*n* = 19 for WT and *n* = 15 for ρ_0 cells). Error bars indicate SD. N.S., not significant. (**C**) The B82 WT cybrids and ρ_0 cells were infected with SeV (5 HAU/mL), and total RNAs from cells were analyzed by RT-PCR to detect viral infection. A region of the SeV *NP gene* (138-386) was amplified and *GAPDH* was used as a loading control. U.I., uninfected. (**D**, **E**) The HeLa WT cybrids and ρ_0 cells were infected

with SeV (5 HAU/mL) for 18 h, and (D) activation of endogenous IRF-3 and (E) the cell-free supernatant was analyzed by ELISA to measure the secreted amount of IFN-β. Error bars indicate SD (*n* = 3). N.D., not detected. U.I., uninfected. In all experiments (A-E), cells were maintained in ρ_0 medium.

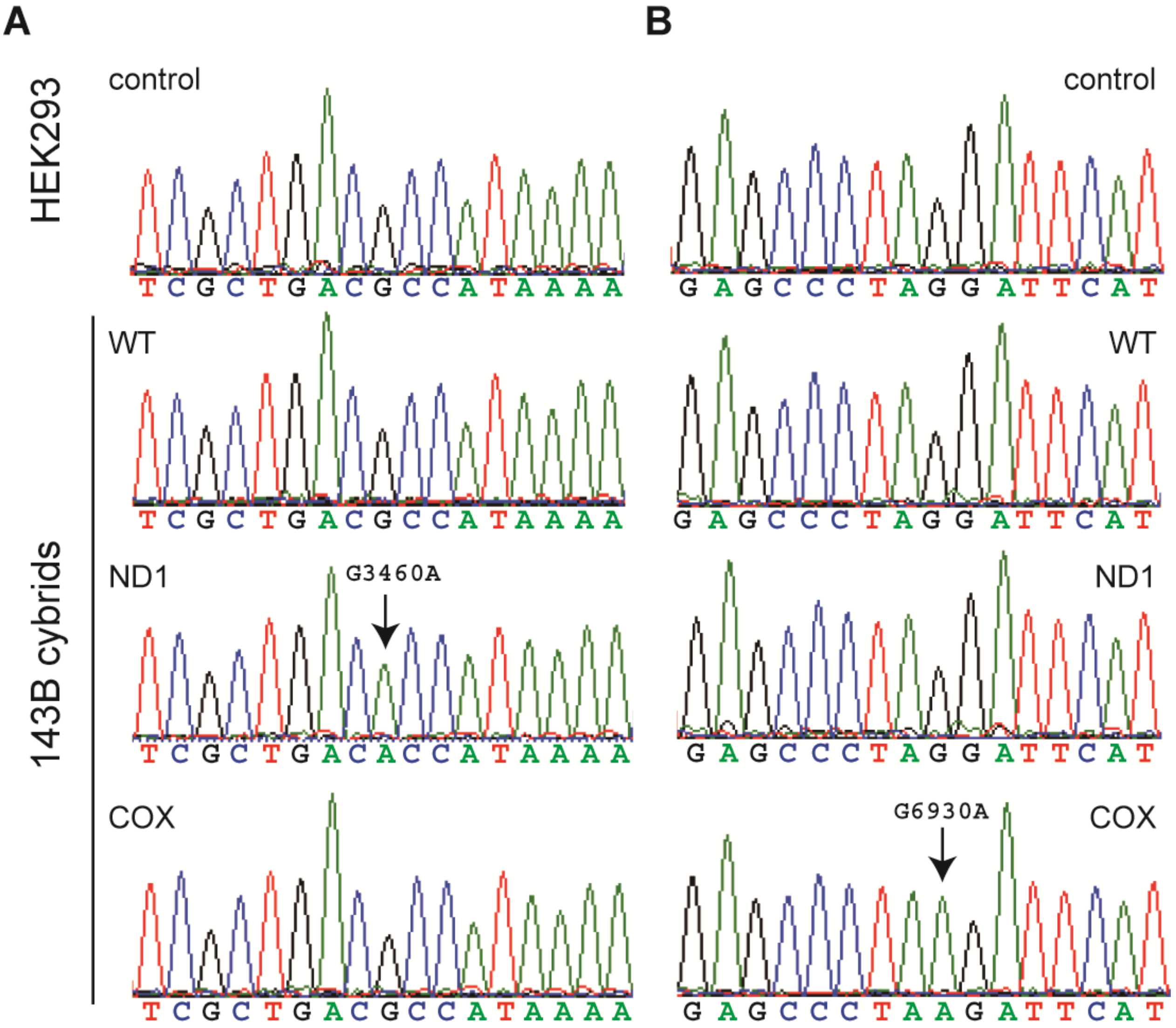

Supplementary Figure S6. Sequential profile of mtDNA mutations in cybrids used in the study. Electropherogram of (**A**) ND1 and (**B**) COX genes amplified from 143B WT, ND1, or COX cybrids as well as HEK293 cells (control) used in the present study. DNA sequencing was performed using forward primers for both the ND1 and COX gene regions. The G-to-A transitions at mtDNA positions 3460 and 6930 are indicated by the arrows.

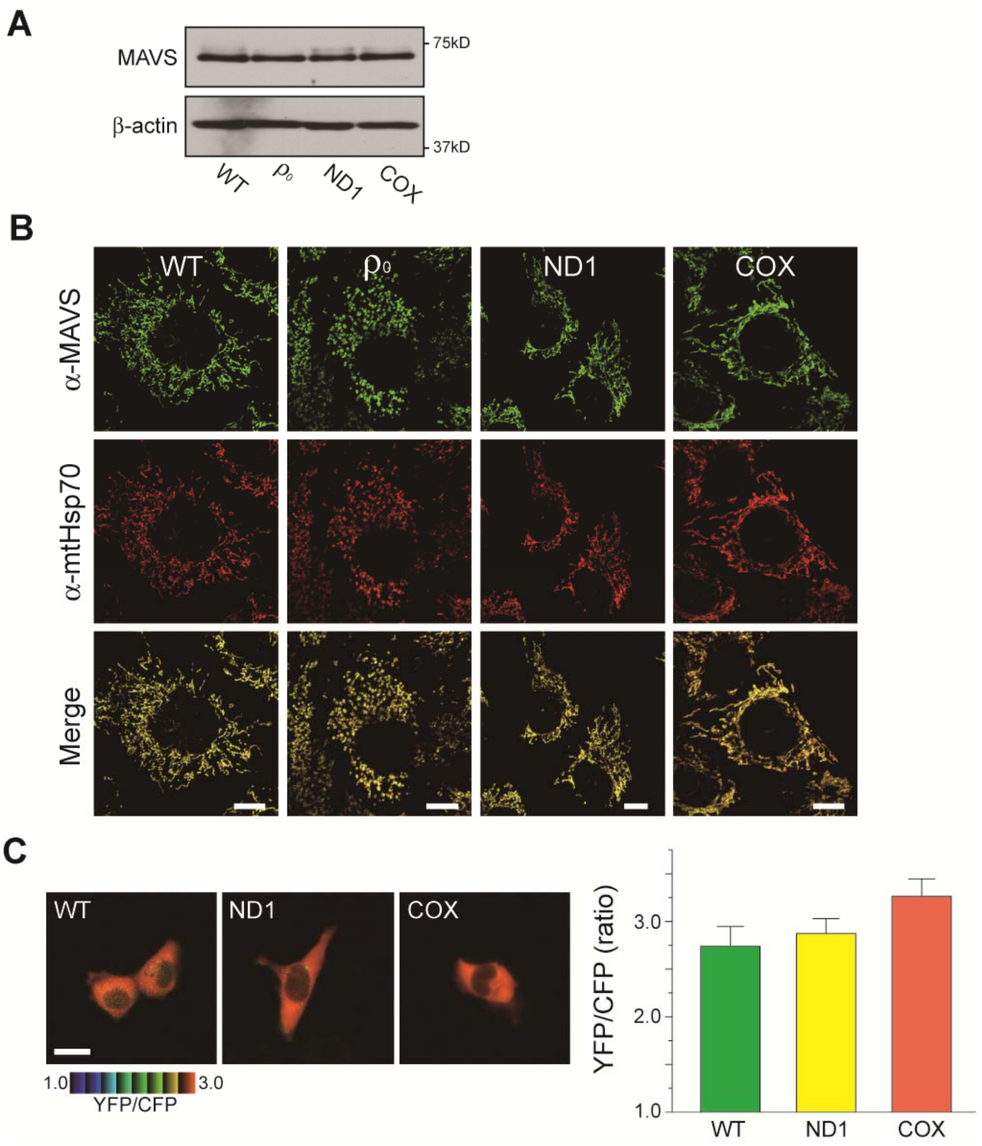

Supplementary Figure S7. Defective RLR pathway in mtDNA mutant cells. (**A**) Western blots revealed the abundance of endogenous MAVS in mtDNA mutant cells. Anti-β-actin was used as the loading control. (**B**) Mitochondrial morphology and MAVS distribution (green) in mtDNA mutant cells (143B WT, ND1, and COX cybrids and its ρ_0 cells) were monitored by immunofluorescence microscopy. Mitochondria in the cells were stained with an anti-mtHsp70 monoclonal antibody (red). Scale bar, 10 μm. (**C**) MtDNA mutant cells expressing ATeam1.03 were cultured in ρ_0 medium, and the time-course of the FRET signal was monitored to visualize the cytoplasmic ATP levels in each living cell. Scale bar, 20 μm. The graph on the right shows the quantification of the YFP/CFP ratio calculated from the images (n = 17 for WT, n = 15 for ND1, and n = 10 for COX). Error bars indicate SD.

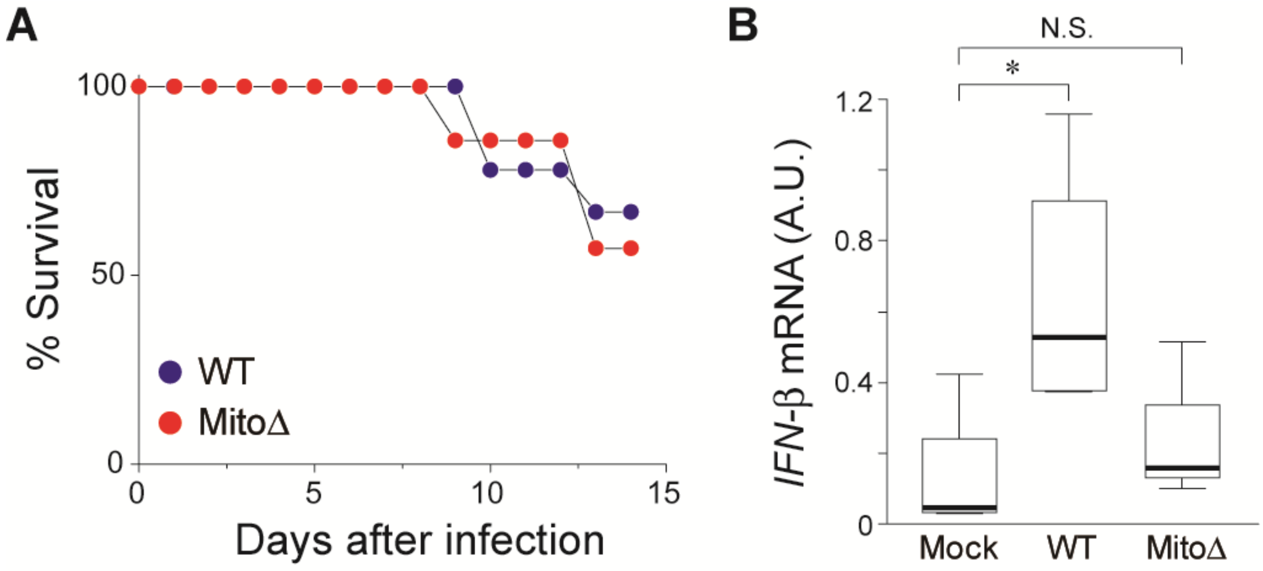

Supplementary Figure S8. Mito-miceΔ are highly susceptible to viral-infection. (**A**) Mito-miceΔ (*n* = 7) or WT mice (*n* = 9) were challenged with IAV (1×10^3 PFU) and mouse survival was monitored for 14 days. (**B**) The IFN-β response was decreased following IAV-infection in lungs of Mito-miceΔ. Quantitative PCR analysis of total RNA isolated from WT (*n* = 4) and Mito-miceΔ (*n* = 3) in lungs 4 days post-infection with IAV. Relative fold-expression levels of *IFN-β* mRNA are shown. As a control, relative mRNA levels of *IFN-β* from mock-infected WT mice (*n* = 5) are also shown. Error bars indicate SD (Unpaired *t*-test; $^*P < 0.05$). N.S., not significant.

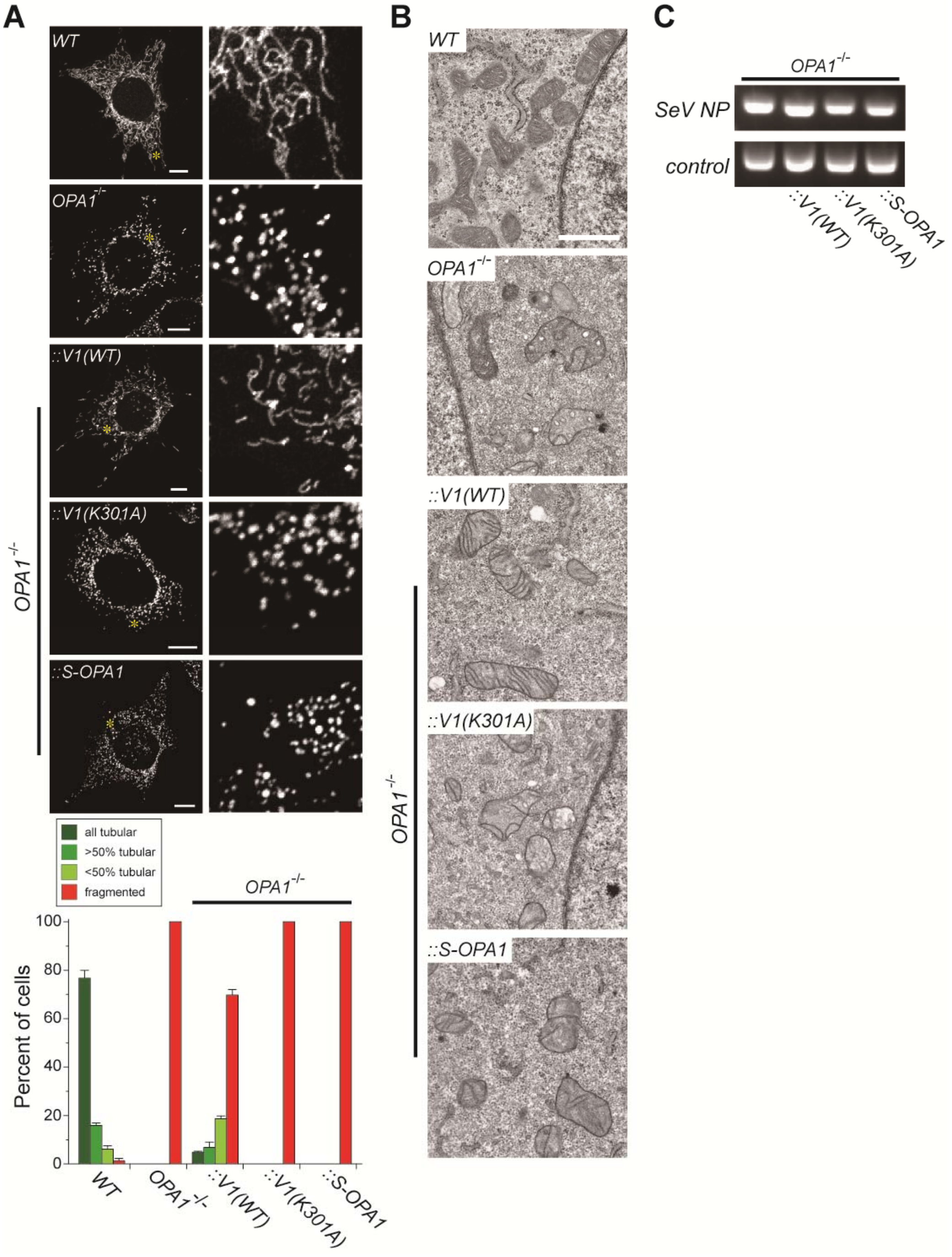

Supplementary Figure S9. Mitochondrial morphology and ultrastructure in *OPA1-/-* and its rescued cells. (**A**) Representative images of mitochondrial morphology in MEFs. Asterisks in the left images denote the area of its magnification enlarged on the right. The graph shows the quantification of the mitochondrial morphology in MEFs. Cells were scored (at least 300 cells) according to one of four morphologic categories, as shown in the inset. Error bars indicate SD. Scale bar, 10 μm. (**B**) The *WT*, *OPA1*$^{-/-}$, and its mutant *OPA1* gene

re-introduced MEFs were analyzed by transmission electron microscopy. Scale bar, 1 μm. (**C**) The *OPA1*$^{-/-}$ and its mutant *OPA1* gene re-introduced MEFs were infected with SeV (4 HAU/mL), and total RNAs from cells were analyzed by RT-PCR to detect viral infection. A region of the SeV *NP gene* (210-386) was amplified and *GAPDH* was used as a loading control.

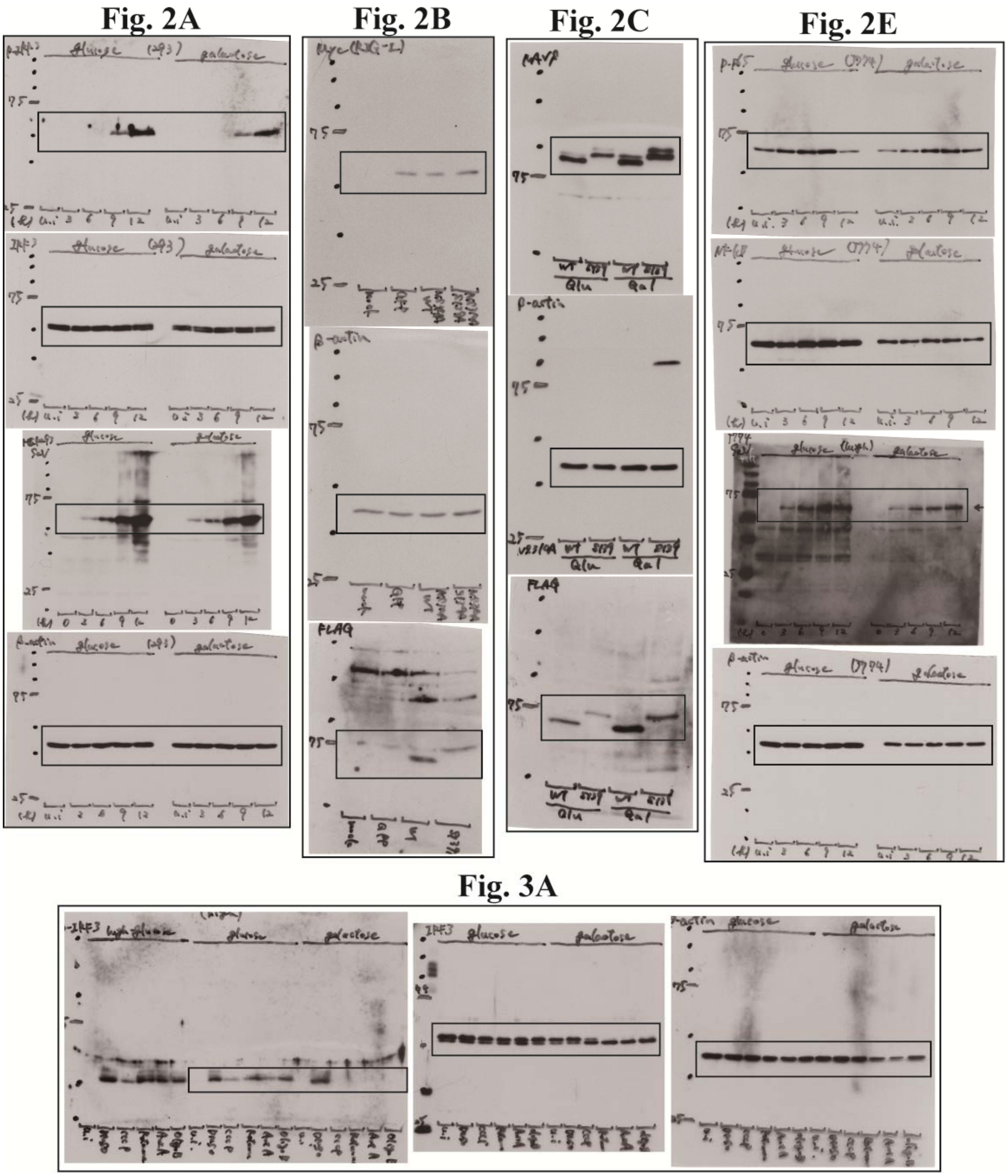

Supplementary Figure S10. Full scan blots from Fig. 2A, 2B, 2C, 2E and 3A. The cropped areas used in the main figures are marked by black boxes.

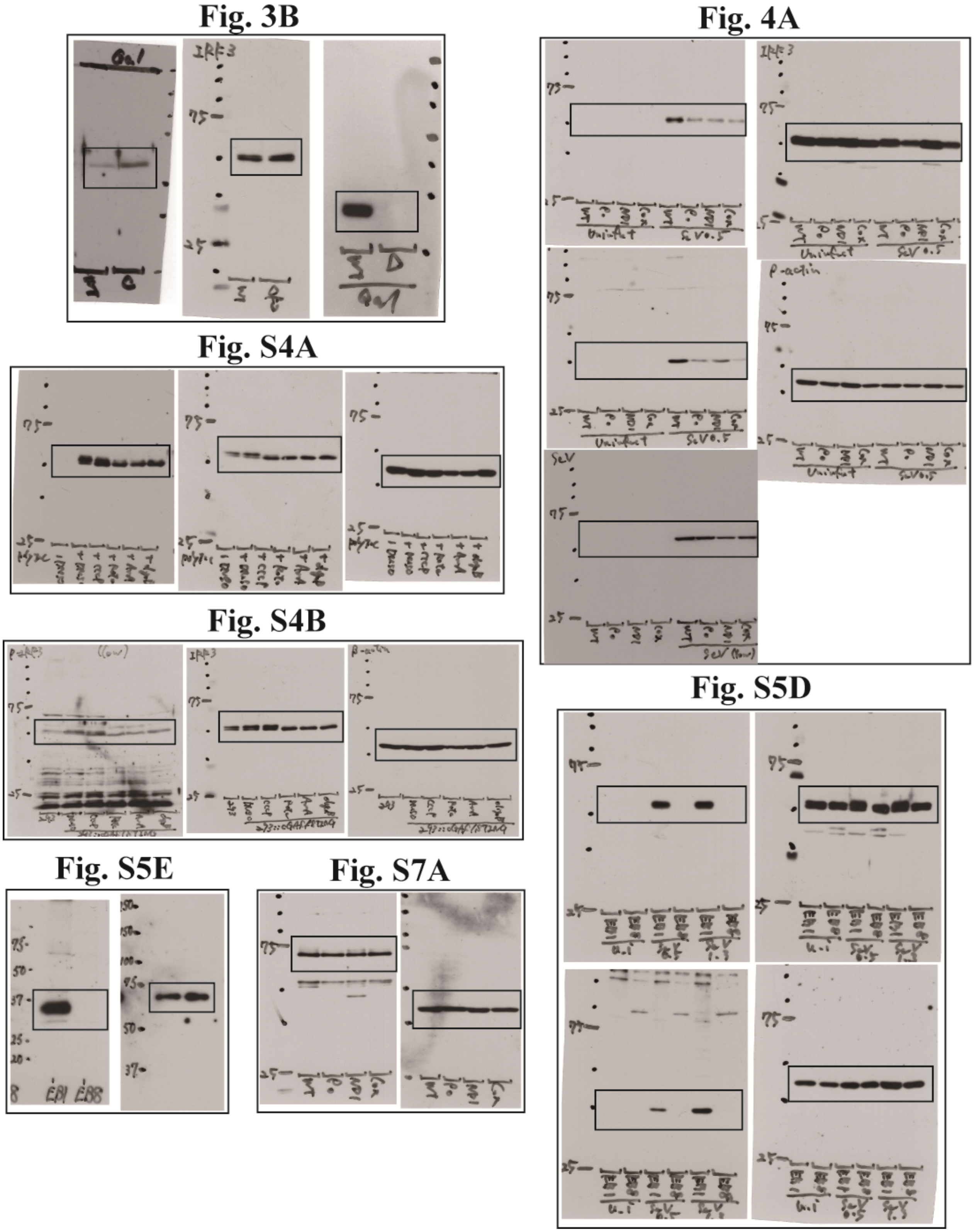

Supplementary Figure S11. Full scan blots from Fig. 3B, 4A, S4A, S4B, S5D, S5E and S7A. The cropped areas used in the main figures are marked by black boxes.
